# Flare hypercalcemia after letrozole in a patient with liver metastasis from breast cancer: a case report

**DOI:** 10.1186/1752-1947-5-495

**Published:** 2011-10-04

**Authors:** Katsumasa Kuroi, Toshinari Yamashita, Tomoyuki Aruga, Kazumi Horiguchi, Dai Kitagawa, Susumu Sekine, Hiromi Tokita, Yuka Hirashima

**Affiliations:** 1 Department of Surgery, Tokyo Metropolitan Cancer and Infectious Diseases Center Komagome Hospital, 3-18-22 Honkomagome, Bunkyo-ku, Tokyo 113-8677, Japan; 2 Department of Pharmacy, Tokyo Metropolitan Cancer and Infectious Diseases Center Komagome Hospital, 3-18-22 Honkomagome, Bunkyo-ku, Tokyo 113-8677, Japan

## Abstract

**Introduction:**

Tamoxifen may occasionally precipitate serious and potentially life-threatening hypercalcemia. However, to date, this has not been documented with aromatase inhibitors.

**Case presentation:**

A 65-year-old Japanese woman with liver metastasis from breast cancer was admitted to our hospital with vomiting, anorexia, fatigue, arthralgia, muscle pain and dehydration. She had started a course of letrozole five weeks earlier. Our patient's calcium level was 11.6 mg/dL. She was rehydrated and elcatonin was administered. Our patient's parathyroid hormone and parathyroid hormone-related protein levels were not increased and a bone scintigram revealed no evidence of skeletal metastasis. After our patient's serum calcium level returned to within the normal range, letrozole was restarted at one-half of the previous dose (1.25 mg). There were no episodes of hypercalcemia. However, 84 days after restarting letrozole, our patient again complained of arthralgia and treatment was changed to toremifene. During these periods, repeated ultrasonograms revealed no progression of liver metastasis.

**Conclusion:**

To the best of our knowledge, this is the first case report of flare hypercalcemia after treatment with letrozole in a patient with metastatic breast cancer.

## Introduction

Flare reaction, a transient exacerbation of symptoms, has been described primarily in breast cancer treatment with tamoxifen and in prostate cancer following therapy with luteinizing hormone-releasing hormone analogues [1,2]. However, the association between a flare reaction and aromatase inhibitors (AIs) has not been documented. We report a case of hypercalcemia that occurred 37 days after initiation of letrozole in a patient with liver metastasis from breast cancer.

## Case presentation

**Figure 1:**
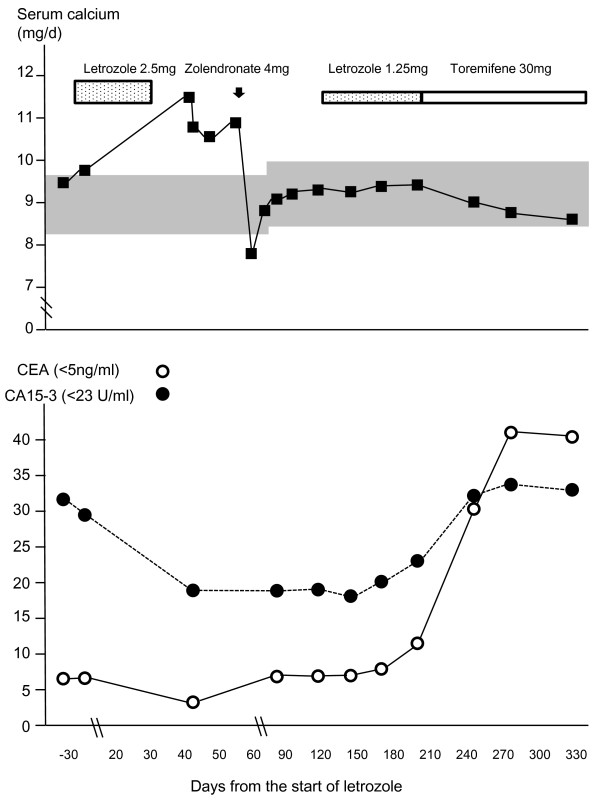
**Changes in serum calcium level and tumor markers before and after flare hypercalcemia**. The normal range of serum calcium level was 8.4 mg/dL to 9.7 mg/dL (this was changed to 8.5 mg/dL to 10.2 mg/dL at midstream), and the calcium level was corrected with albumin by a standard calculation.

A 65-year-old Japanese woman was admitted to our hospital one year ago with vomiting, anorexia, fatigue, arthralgia, muscle pain, and dehydration (Figure 1). Our patient had undergone a right mastectomy 30 years previously and received adjuvant chemoendocrine therapy (doxifluridine and tamoxifen) without complications. Five years after that surgery, she developed a tumor in her liver and a needle biopsy revealed metastatic adenocarcinoma from breast cancer (estrogen-receptor positive, progesterone-receptor positive, Her2 negative). Since then, our patient has been treated with taxanes and capecitabine, followed by doxifluridine and medroxyprogesterone acetate. Using doxifluridine and medroxyprogesterone acetate, she remained well and achieved a complete response without an increase of carcinoembryonic antigen (CEA) or carbohydrate antigen (CA) 15-3 for eight years. However, three months before this current admission, CEA and CA 15-3 had increased to 6.3 ng/mL (normal value < 5 ng/mL) and 30.6 IU/mL (normal value < 23 IU/mL) respectively and an abdominal ultrasonogram revealed recurrence of liver metastasis. A computed tomography (CT) scan was normal. Letrozole was initiated with alendronate (T score -2.8) and withdrawn three weeks later due to severe muscle pain and arthralgia. Two weeks after the onset of these symptoms, the severity increased and our patient was admitted to our hospital. Her serum calcium level was 11.6 mg/dL. She was rehydrated and elcatonin was administered. Parathyroid hormone (PTH) and parathyroid hormone-related protein (PTHrP) levels were not increased and a bone scintigram, CT and thoracolumbar survey revealed no evidence of skeletal metastasis. Intravenous bisphosphonate was not administered as our patient had been undergoing dental treatment. She was discharged when symptoms subsided on day 11. One week later, after the completion of her dental treatment, our patient was administered zoledronate for persistent hypercalcemia. Thereafter, our patient was readmitted due to hypocalcemia. After our patient's serum calcium levels returned to within normal range, letrozole was restarted at one-half dose (1.25 mg) with no reoccurrence of hypercalcemia. Unfortunately, 84 days after the restart, letrozole was withheld due to intolerable arthralgia and our patient's therapy was changed to toremifene. Although she complained of mild arthralgia while on toremifene, the symptom gradually subsided and she has remained well. Repeated ultrasonograms revealed no progression of liver metastasis. Our patient's CEA and CA 15-3 have again increased to pretreatment levels but have remained stable.

## Discussion

A flare reaction was first observed during the treatment of postmenopausal women with high-dose estrogen and has been frequently documented with tamoxifen [1,3]. This reaction comprises two different manifestations: tumor flare and flare hypercalcemia [2]. The former includes an increase in swelling, erythema, itching, or pain in soft-tissue metastasis, the development of new lesions, an increase of tumor markers and an increase in skeletal pain in patients with bone metastasis. Tumor flare can be accompanied by flare hypercalcemia.

The distinction between spontaneous hypercalcemia and flare hypercalcemia is sometimes difficult to ascertain. Although both occur most often in patients with widespread bone metastases [4,5], flare hypercalcemia can be seen in patients without apparent bone involvement [6,7], as in the case of our patient. Moreover, flare hypercalcemia has a rapid onset that characteristically occurs within several days of starting therapy; symptoms are usually short-lived and serum calcium levels return to normal when the offering agent is withdrawn. In contrast, spontaneous hypercalcemia is generally slow in onset and, accordingly, symptoms develop gradually [1,8]. In general, the following three criteria are used to prove a causal relation between drug and symptom: the symptom should develop after drug administration, reverse when it is withdrawn, and be reinduced by rechallenge [9]. In this respect, flare hypercalcemia could meet two of these three criteria. Similar to other reports of flare hypercalcemia [5,6,8-11], the third criterion is not met in the case of our patient; however, the temporary relation of the onset of hypercalcemia and administration of letrozole strongly suggests a causal role for the drug.

Our review of the literature did not reveal any description of flare hypercalcemia by AIs with the exception of one report of tumor flare and tumor lysis syndrome by letrozole. In a study by Zigrossi *et al*. [12], a 61-year-old woman experienced tumor flare (pleural and pericardial effusion) and tumor lysis syndrome on the second day of letrozole administration and subsequently recovered from critical condition, notwithstanding continuation of letrozole. Tumor lysis syndrome is characterized by the rapid death of neoplastic cells that develops soon after effective therapy. The biochemical and clinical features of this syndrome include hyperazotemia, hyperuricemia, hyperkalemia, hypocalcemia, elevated lactate dehydrogenase, hypotension and acute renal failure. The patient in the study did not develop hypercalcemia.

The exact mechanism of flare hypercalcemia remains uncertain, and it is believed that estrogenic properties of tamoxifen may precipitate hypercalcemia in tamoxifen-induced hypercalcemia. Although this does not account for AIs, it is interesting to note that flare hypercalcemia could occur in estrogen-receptor negative patients [9]. Thus, it might be plausible to consider that flare hypercalcemia is due to increased osteoclast activities and bone resorption caused by the increased release of various factors from the tumor or host cells by the offending drug [7,11,13]. Importantly, normal PTH and/or PTHrP levels in our patient exclude the possibility of coexisting primary hyperparathyroidism and ectopic secretion of PTH and/or PTHrP caused by tumor cells. Another cause of hypercalcemia might include inappropriately increased production of 1, 25-hydroxyvitamin D3 typically seen in patients with lymphomas or sarcoidosis [7]. However, the vitamin D3 levels were not assessed and CT and clinical findings did not suggest the possibility of these conditions as a cause of flare hypercalcemia in our patient.

It is difficult to predict and to prevent flare hypercalcemia, but life-threatening hypercalcemia should be treated intensively by stopping the offending drug, rehydration and bisphosphonate treatment [13,14]. As for rechallenge of the offending drug, it is usually suggested that, if necessary, tamoxifen be resumed temporarily at a lower dosage in tamoxifen-induced hypercalcemia. This is because tumor regression could occur after the flare reaction subsides and the survival of patients with tamoxifen-induced hypercalcemia is reported to be longer than that of patients with spontaneous hypercalcemia [1,2,8,15]. This strategy also appears to apply to AIs [12].

## Conclusion

Our experience suggests that AIs may precipitate serious and potentially life-threatening hypercalcemia in the early stages of treatment. If this occurs, AIs could be restarted cautiously with therapeutic benefit. To our knowledge, the association of hypercalcemia and AIs has not been previously reported.

## Consent

Written informed consent was obtained from the patient for publication of this case report and any accompanying images. A copy of the written consent is available for review by the Editor-in-Chief of this journal.

## Competing interests

The authors declare that they have no competing interests.

## Authors' contributions

TY, TA, KH, DK, SS, HT analyzed and interpreted the patient data regarding the hypercalcemia. YH collected the clinical data and reviewed the literature about flare. KK was the doctor in charge and was a major contributor in writing the manuscript. All authors read and approved the final manuscript.
